# Gα_i_-mediated TRPC4 activation by polycystin-1 contributes to endothelial function via STAT1 activation

**DOI:** 10.1038/s41598-018-21873-1

**Published:** 2018-02-22

**Authors:** Misun Kwak, Chansik Hong, Jongyun Myeong, Eunice Yon June Park, Ju-Hong Jeon, Insuk So

**Affiliations:** 1 Department of Physiology and Institute of Dermatological Science, Seoul National University College of Medicine, Seoul, 110-799 South Korea; 2 Department of Biomedicines, Seoul National University College of Medicine, Seoul, 110-799 South Korea; 3 Department of Physiology, School of Medicine, Chosun University, Gwangju, 61452 South Korea

## Abstract

Hypertension and aneurysm are frequently associated with autosomal dominant polycystic kidney disease (ADPKD) caused by polycystin-1 (PC1) mutations, which is closely related to endothelial dysfunction. PC1 is an atypical G-protein-coupled receptor that activates G-proteins by self-cleavage; currently, however, the molecular and cellular mechanisms of the associated intracellular signaling and ion channel activation remain poorly elucidated. Here, we report an activation mechanism of a calcium-permeable canonical transient receptor potential 4 (TRPC4) channel by PC1 and its endothelial function. We found that the inhibitory Gα_i3_ protein selectively bound to the G-protein-binding domain on the C-terminus of PC1. The dissociation of Gα_i3_ upon cleavage of PC1 increased TRPC4 activity. Calcium influx through TRPC4 activated the transcription factor STAT1 to regulate cell proliferation and death. The down-regulation of PC1/TRPC4/STAT1 disrupted migration of endothelial cell monolayers, leading to an increase in endothelial permeability. These findings contribute to greater understanding of the high risk of aneurysm in patients with ADPKD.

## Introduction

Autosomal dominant polycystic kidney disease (ADPKD) is one of the most common inherited diseases. ADPKD is characterized by the progressive expansion, in both kidneys, of multiple fluid-filled cysts, which gradually replace normal renal tissue and ultimately result in end-stage renal failure^1^. In ADPKD-causative genes, the *PKD1* gene, which encodes polycystin-1 (PC1) and accounts for 85% of all cases, is involved in the control of epithelial cell population growth^2–4^, migration^5,6^, differentiation^7^ and apoptosis^8^. In addition, PC1 is required for regulation of the cell cycle^9^ and activation of cation-permeable currents^10–12^ by regulation of G-protein signaling^13,14^.

PC1 is a glycoprotein that consists of approximately 4,302 amino acids, weighs approximately 460 kDa and has 11 transmembrane domains, with a huge N-terminal extracellular region including the G protein-coupled receptor (GPCR) proteolytic site (GPS) motif. Previous studies have suggested that PC1 functions as an atypical GPCR, binds heterotrimeric Gα_i/o_ proteins and regulates calcium flux through PC2 (TRPP2) by releasing Gβγ subunits. In addition, N-terminal cleavage of PC1 at the GPS motif promotes the formation of various C-terminal fragments or tails (CTFs or CTTs), which modulate diverse signaling pathways via translocation to the nucleus^15^. In addition, missense mutations in the GPS disrupt the cleavage of PC1 and prevent activation of the JAK-STAT pathway^16^. The dissociation of PC1 from the N-terminal fragment (NTF) or CTF is related to disturbed intracellular Ca^2+^ homeostasis and cAMP accumulation, leading to abnormal cell proliferation and the growth of multiple cysts^17^. Thus, calcium and cAMP are important regulatory players in the cell biology of PKD.

Transient receptor potential (TRP) channels make up a family of seven cationic channels, which are divided into 7 subfamilies based on amino acid similarity. TRP channels can form functional homo- or hetero-tetrameric channels with intra-subgroups or even with inter-subfamilies^18^. The polycystic type of TRP (TRPP) channel is associated with polycystic kidney disease, which results from abnormal Ca^2+^ homeostasis and signaling^19^. Newby *et al*.^20^ suggested that the TRPP2/PC1 receptor-ion channel complex plays a critical role in renal physiology. The classical TRP (TRPC) is a receptor-operated channel (via G protein-coupling), which is primarily activated in response to PLC activation^21^ or inhibitory Gα (Gα_i_) interactions^22^. By inducing dissociation of heterotrimeric Gα_i/o_/βγ proteins by PC1, we hypothesized that PC1 could activate the TRPC4 channel via Gα_i/o_.

Vascular endothelial cell Ca^2+^ entry through TRPC4 leads to vascular smooth muscle relaxation in an endothelial acetylcholine-dependent manner, as well as endothelial hyperpermeability via disruption of cell junction complexes or cytoskeletal reorganization^23^. Cerebral aneurysms are more common in ADPKD patients with loss of function or missense mutations in the PC1-encoding *PKD1* gene^24–26^. Accordingly, the functional interaction of TRPC4 with PC1 is essential for Ca^2+^ regulation in the endothelium, but their pathophysiological mechanisms remain unknown.

In the present study, we overexpressed recombinant TRPC4 and PC1 in HEK cells and analyzed ion channel activity using electrophysiological techniques and Ca^2+^-dependent signaling using molecular biological methods. Furthermore, we confirmed the functional interaction of TRPC4 with PC1 in HUVECs. Taken together, these findings are consistent with Gα_i_-dependent TRPC4 activation by the PC1 protein. The Gα_i_ dissociation by GPS cleavage of PC1 activates TRPC4, which in turn elevates Ca^2+^ levels available for triggering the activation of STAT1 in endothelial cells.

## Results

### Identification of polycystin-1 (PC1)

**Figure 1:**
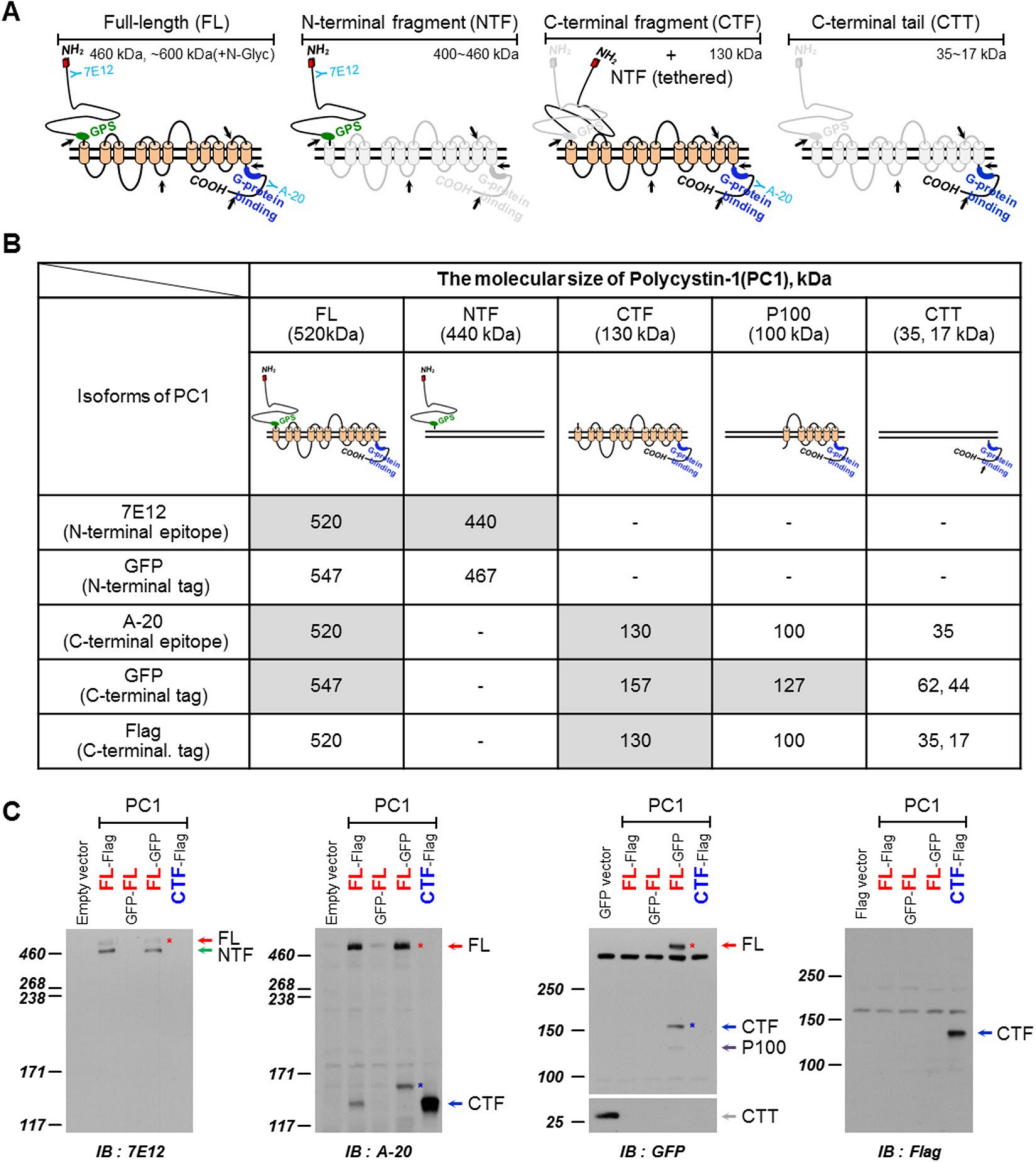
Schematic diagram of PC1 structure and identification of PC1 products. (**A**) Schematic structure of human PC1. FL, full-length; NTF, N-terminal fragment; CTF, C-terminal fragment; GPS, G protein-coupled receptor proteolytic site. PC1 cleavage occurs at the GPS motif, resulting in NTF and CTF fragments. At least three proteolytic cleavages occur in or near the C-terminal tail that result in the release of protein fragments. The possible cleavage sites are indicated by arrows. A blue color indicates an epitope recognized by an anti-PC1 antibody (7E12 or A-20). (**B**) Recognized cleavage forms of PC1 by antibodies. (**C**) Validation of *PKD1* constructs and identification of PC1 cleavage products by over-expression with Flag or GFP-tagged PC1(FL or CTF), tagged at either the N- or C-terminus. The proteins were detected by an anti-PC1 (7E12) antibody (*first panel*). FL (*red arrow or red star*) and NTF forms (*green arrow*) of PC1 were observed with FL-Flag and FL-GFP. The cleaved CTF forms (*blue star*) and non-cleaved FL (*red star*) were detected by an anti-PC1 (A-20) antibody (*second panel*). Using an anti-GFP antibody (*third panel*), FL (*red star*), CTF (*blue star*), and P100 of FL-GFP were detected, but not CTT. The cleaved CTF forms (*blue arrow*) were detected by an anti-Flag antibody (*fourth panel*). HEK 293 cells were transiently transfected with empty vector (pcDNA3, GFP, or Flag, *lane 1*), FL-Flag (*lane 2*), GFP-FL (*lane 3*), FL-GFP (*lane 4*), and CTF-Flag (*lane 5*).

Polycystin-1 (PC1) is a large plasma membrane glycoprotein that undergoes several proteolytic cleavages, including autocatalytic cleavage at the G protein-coupled receptor proteolytic site (GPS) (Fig. 1). The cleaved PC1 consists of an N-terminal fragment (NTF) associated with a C-terminal fragment (CTF). At least three other cleavages liberate portions of the cytoplasmic C-terminal tail (CTT) of PC1 (Fig. 1A,B). To determine PC1 expression patterns, we used variously tagged (e.g., GFP, Flag, and HA) constructs at each terminus of PC1 and performed Western blot analysis with several detection antibodies using lysates of HEK293 cells expressing human *PKD1* (Fig. 1B). Using an antibody against the PC1 N-terminus (7E12), two bands at ≥460 kDa, full-length (FL) at 520 kDa and NTF at 440 kDa, were observed in a PC1 construct flag-tagged at the N terminus (PC1-Flag (FL-Flag)) and a PC1 construct GFP-tagged at the C terminus (PC1-GFP (FL-GFP)) (Fig. 1C, Supplementary Fig. 9). With an antibody against the PC1 C-terminus (A-20), FL and CTF (130 kDa) were detected in PC1-Flag, and FL-GFP and CTF-GFP (157 kDa), a cleavage product of *hPC1-GFP*, were detected in PC1-GFP (Fig. 1C, Supplementary Fig. 9). With an antibody against GFP (GFP), FL-GFP and CTF-GFP were detected in PC1-GFP. With an antibody against Flag (Flag), CTF was detected in CTF-Flag.

### Characterization of PC1 as a G protein-coupled receptor (GPCR)

**Figure 2:**
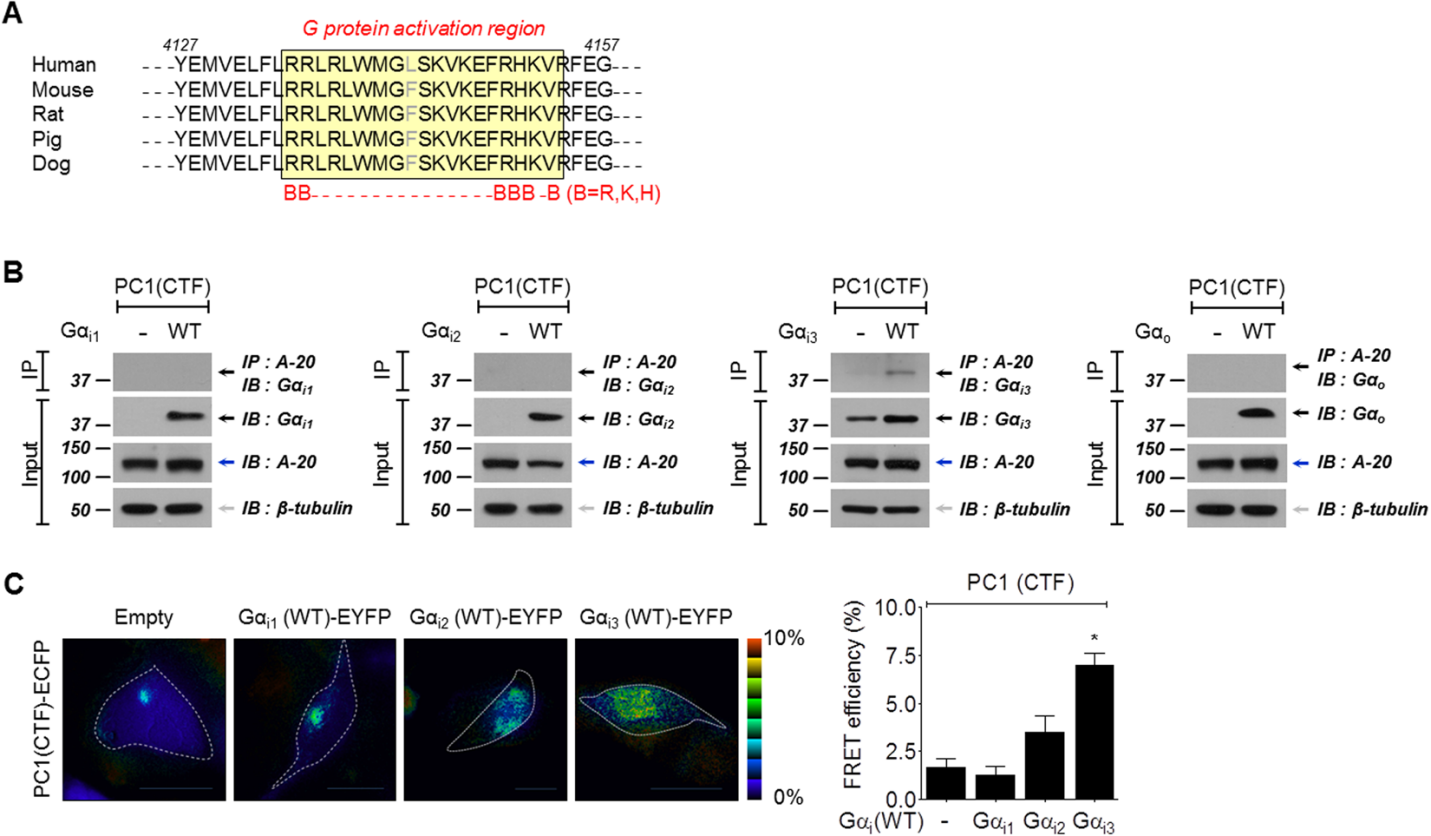
Interaction of PC1(CTF) with Gα_i3_. (**A**) Alignment of amino acid sequences of the PC1 C-terminal cytoplasmic tail across many species. The G protein activation region of the PC1 C-terminal tail is conserved across many species (residues 4135-4154 of human PC1). This region is highlighted by the yellow shading. (**B**) Interactions between PC1(CTF) and Gα_i/o_ subtypes. Gα_i/o_ subtypes and PC1(CTF) were co-expressed in HEK 293 cells. 500 μg of proteins from each condition were subjected to immunoprecipitation with anti-A-20 and probed with an antibody against Gα_i/o_ proteins. PC1(CTF) interacts directly with Gα_i3_ but not with other Gα_i/o_ subtypes. (**C**) FRET-detectable interactions between PC1(CTF) and Gα_i_ subtypes. Representative FRET images of hPKD1(CTF)-ECFP co-expressed with Gα_i1_(WT)-, Gα_i2_(WT)-, and Gα_i3_(WT)-EYFP compared to empty vector (pEYFP-N1) expression. A bar graph of FRET efficiency between PC1(CTF) and Gα_i_ subtypes.

A fundamental feature of PC1 is post-translational modification via cleavage at the juxtamembrane GPCR proteolysis site (GPS) motif that is part of the larger GAIN domain^27^. The PC1 C-terminal cytosolic domain also has a G protein activation region, which is defined as a sequence of ≤25 amino acids with the consensus motif BB…..BBxB or BB…..BBxxB (B = R, K, or H) (Fig. 2A). Signaling pathways of PC1 may be mediated by activating or binding to heterotrimeric G proteins^13^. Therefore, PC1 may act as an atypical GPCR, which belongs to the GPCR family with GAIN domain.

To identify binding between PC1 and specific Gα protein subunits, we heterologously expressed CTF of PC1 (PC1(CTF)) and Gα subunits in HEK293 cells and analyzed them via co-immunoprecipitation (Co-IP) and FRET imaging (Fig. 2). The CTF of PC1 has a C-terminal cytoplasmic tail (CTT), including a G protein activation region and a coiled-coil domain. The CTF of PC1 was used because the N-terminal extracellular region does not participate in cleavage signaling. Protein was immunoprecipitated with anti-A-20 and then probed with anti-Gα antibodies. The IP band was observed only for the co-expression of PC1(CTF) with Gα_i3_, indicating that PC1 specifically interacts with Gα_i3_ (Fig. 2B, Supplementary Fig. 10). The FRET efficiency between PC1(CTF) and Gα_i_ was also measured. When we co-transfected CFP-tagged PC1(CTF) and YFP-tagged Gα_i_, the highest FRET efficiency was recorded between PC1(CTF) and Gα_i3_ (Fig. 2C). Next, the interaction between full-length PC1 (PC1(FL)) and Gα_i3_ was investigated (Supplementary Fig. 1A). The binding was weaker than for PC1(CTF) (Supplementary Fig. 1C). A GPS site deletion mutant (ΔGPS) was also used to evaluate the interaction with Gα_i3_ (Supplementary Fig. 1B). Other G proteins, e.g., Gα_s_, Gα_q_, and Gα_12_, did not bind with PC1(CTF) (Supplementary Fig. 2A). These results suggest that PC1 is able to bind to specific G protein subunits and likely acts as a GPCR in cells and tissues.

### The activation of the TRPC4β channel by PC1 cleavage

**Figure 3:**
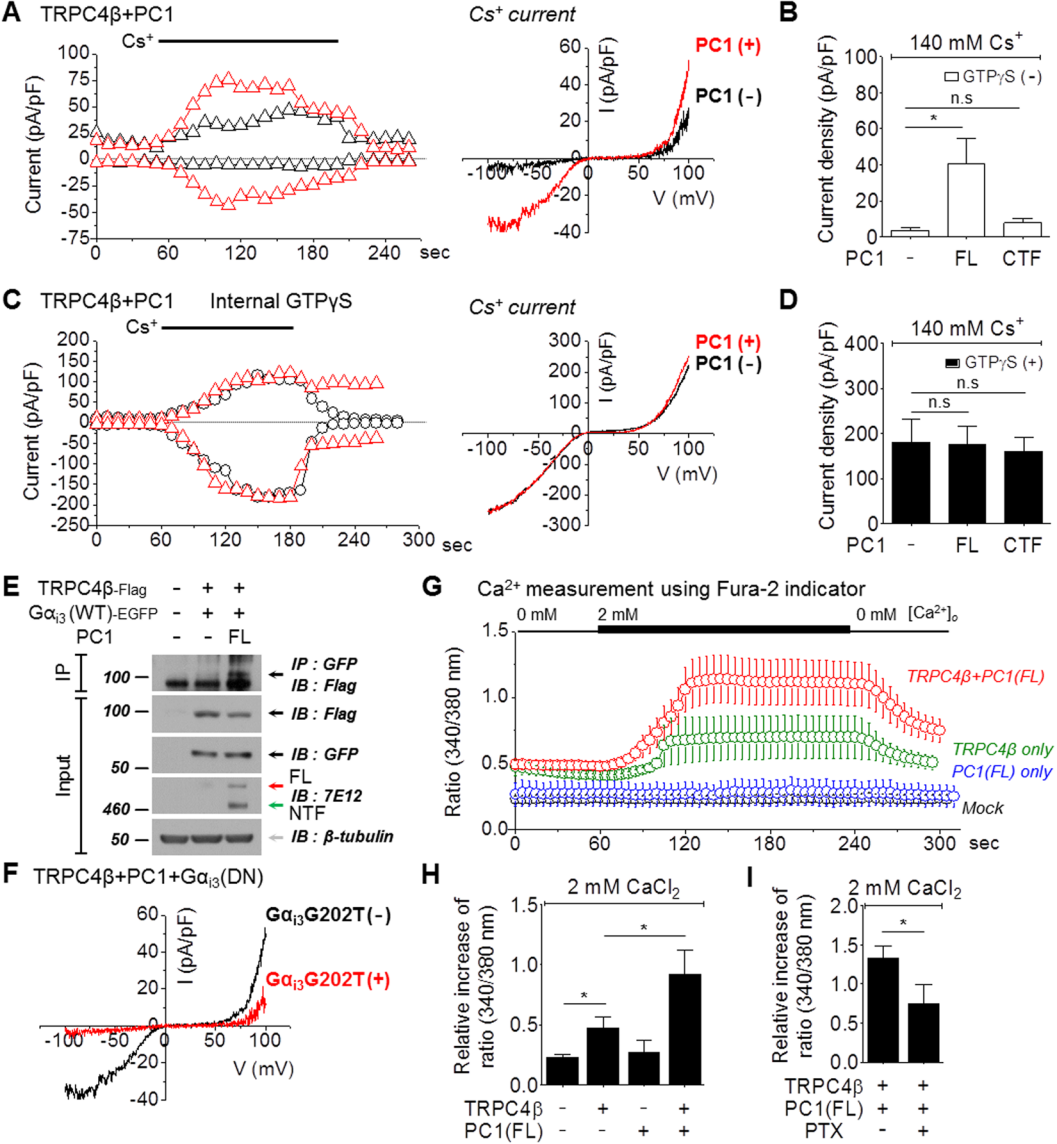
Activation of TRPC4β by PC1(FL). (**A**) HEK 293 cells were co-transfected with TRPC4β and PC1(FL) or PC1(CTF). The TRPC4 current was recorded without GTPγS, and the current amplitude was increased by 140 mmol/L Cs^+^. The current amplitudes at −60 mV and +60 mV were plotted against time (the left panel) in HEK cells expressing PC1 and TRPC4β (red triangle) or TRPC4β (black triangle). The ramp pulses were applied every 10 sec. The I-V curves from HEK cells expressing PC1 and TRPC4β (red line) or TRPC4β (black line) showed a double-rectifying shape. (**B**) The bar graphs represent the means ± s.e.m of current density (pA/pF) at −60 mV in the absence of GTPγS infusion. (**C**) The current was recorded in TRPC4β/PC1 co-transfected HEK 293 cells infused with GTPγS. The I-V curves from HEK cells expressing PC1 and TRPC4β (red line) or TRPC4β (black line) showed a double-rectifying shape. (**D**) The bar graphs represent the means ± s.e.m of current density (pA/pF) at −60 mV in the presence of GTPγS infusion. (**E**) The interactions between TRPC4β and Gα_i3_ subtypes in the presence or absence of PC1(FL). (**F**) Inhibition of PC1-mediated TRPC4β activity by a dominant-negative Gα_i3_. HEK 293 cells were transfected with TRPC4β, PC1(FL), and Gα_i3_ G202T (dominant-negative Gα_i3_). The I-V relationship is shown before (black) and after maximal current (red) inhibition. (**G**) [Ca^2+^]_i_ measurements in PC1-mediated activity of TRPC4β. Cytoplasmic calcium measurements in cells expressing TRPC4β alone or co-expressing PC1(FL) and TRPC4β loaded with Fura-2 (ratiometric measurement at 340 nm and 380 nm, expressed as 340/380). The cells were perfused with an extracellular solution containing no added calcium, and then extracellular calcium was increased to 2 mmol/L for 3 minutes. (**H**) Bar graph showing the increase in calcium influx in TRPC4β channels activated by PC1(FL). (**I**) Bar graph showing the inhibition in pretreatment with 100 ng/ml PTX. **p < 0.01, *p < 0.05 and n.s. not significant.

Since PC1 is considered a GPCR, albeit an atypical one, and TRPC4β can be activated by GPCR, we investigated whether PC1 affects TRPC4β channel currents. The TRPC4β channels expressed in HEK293 cells had lower basal activity. The measurements of TRPC4β activity could be manipulated by altering the extracellular ion composition. To efficiently measure TRPC4β activity, we used 140 mmol/L Cs^+^-rich solution on the basis of the high permeability of Cs^+^ ions in TRPC4. The amplitude of TRPC4 currents was 4 ± 1 pA/pF (n = 7) in 140 mmol/L Cs^+^-rich solution. When TRPC4β was co-expressed with PC1(FL), TRPC4β current was increased by PC1(FL) (41 ± 14 pA/pF, n = 7) under Cs^+^-rich conditions (Fig. 3A,B). The co-expression of TRPC4β with PC1(CTF) did not increase TRPC4β currents (7 ± 3 pA/pF, n = 3) compared with controls (Fig. 3B). To activate TRPC4β, we used intracellular GTPγS, which activates different types of G proteins, through a patch pipette. Intracellular GTPγS administration fully activated TRPC4β. GTPγS-induced Cs^+^ currents in TRPC4β by PC1(FL) or PC1(CTF) showed no differences compared with controls (Fig. 3C,D). We investigated whether Gα_i3_ that was dissociated from CTT by cleavage of PC1 affected TRPC4β activity. We observed the interaction of TRPC4β with Gα_i3_ protein in both the presence and absence of PC1(FL) using Co-IP. Expression of PC1(FL) significantly increased their physical interaction (Fig. 3E, Supplementary Fig. 11). TRPC4β current was inhibited by a dominant negative form of Gα_i3_ protein (Gα_i3_ G202T) in HEK cells transfected with PC1(FL) (Fig. 3F). To determine whether PC1 constitutively activates the TRPC4 channel, we performed Ca^2+^ measurements and analyzed the relative increase in calcium. PC1(FL) significantly increased TRPC4β-mediated Ca^2+^ influx when the external Ca^2+^ was increased from 0 mmol/L to 2 mmol/L (Fig. 3G,H). The pretreatment of 100 ng/ml PTX partially attenuated the relative increase of Ca^2+^ in HEK cells co-expressed with PC1 and TRPC4β (Fig. 3I). Finally, we investigated whether PC1 increases TRPC4β currents by regulating channel expression. PC1 did not increase the surface expression of TRPC4β channel (Supplementary Fig. 3). These results suggest that the activation of TRPC4β by PC1 is mediated by Gα_i3_, leading to increased intracellular Ca^2+^ levels.

**Figure 4:**
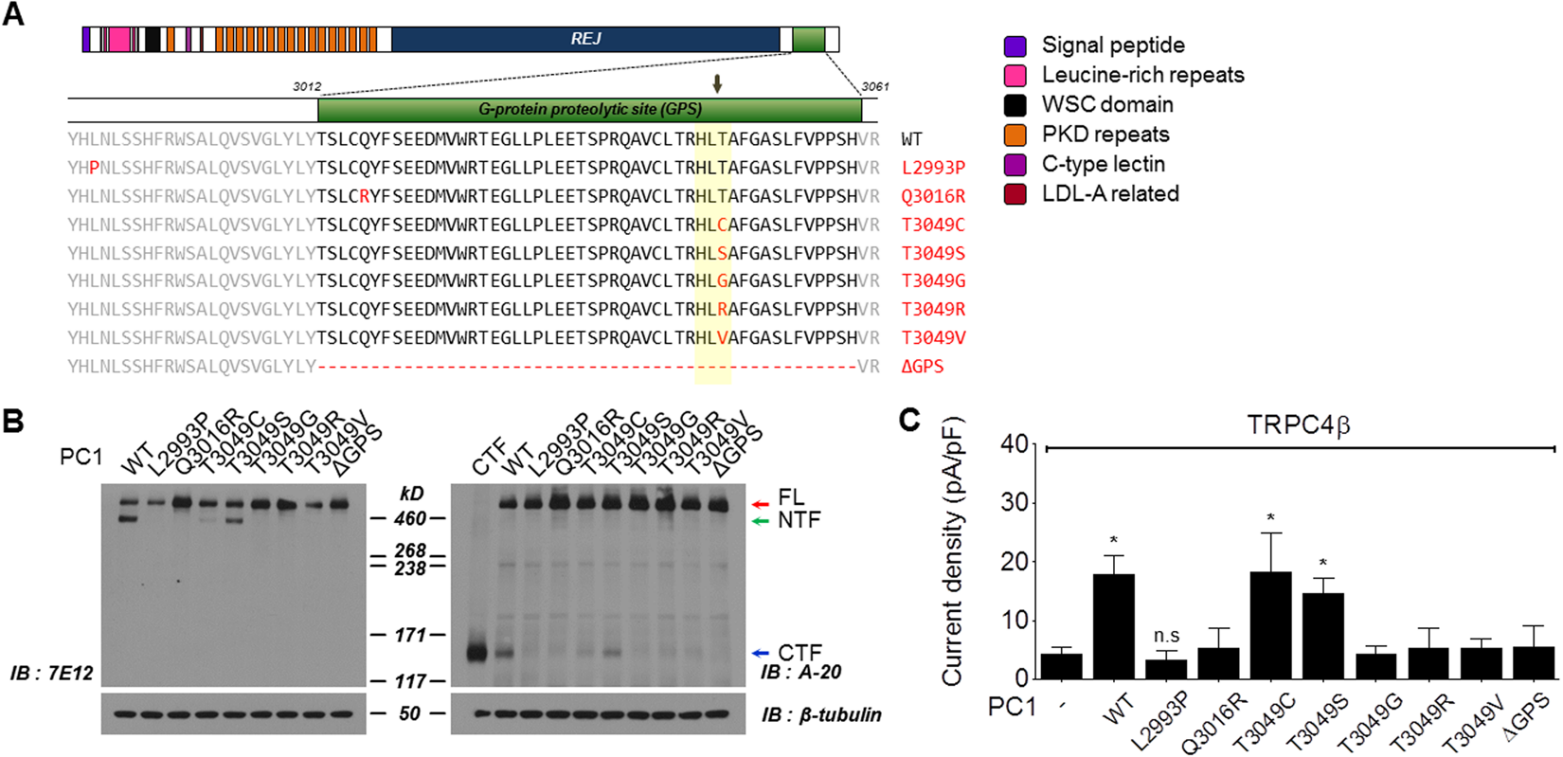
Effects of amino acid substitutions on proteolytic cleavage of PC1. (**A**) Schematic structure of the PC1 N-terminal domain. All domains are shown by colored boxes. PC1 cleavage occurs at the HL↓T^3049^ site in the GPS domain. The cleavage site is marked by an arrow. Two germline mutants (L2993P and Q3016R), substitution of 3049 Thr (T) by Cys (C), Ser (S), Gly (G), Arg (R), and Val (V), and deletion of the GPS domain were generated using QuickChange site-directed mutagenesis. (**B**) HEK 293 cells were transfected with *hPC1(FL)-flag* constructs containing missense mutations and assayed for cleavage as indicated. Cell lysates were immunoblotted with anti-7E12 (*left*) and anti-A-20 (*right*). Mutant T3049C and T3049S conserved cleavage of PC1 at the GPS site. (**C**) Activity of TRPC4β by non-cleavable PC1 mutants. HEK 293 cells were transfected with TRPC4β and wild-type or non-cleavable mutants of PC1(FL). The bar graphs represent the means ± s.e.m. of current density (pA/pF) at −60 mV of the indicated experiments. *p < 0.05 and n.s. not significant.

PC1 is constitutively cleaved at His-Leu↓Thr3049 (between Leu3048 and Thr3049) within the GPS domain^28,29^. The most well-characterized mechanism is the *cis*-autoproteolysis, a self-catalyzed protein rearrangement that results in cleavage at HX↓(T/S/C)^30^, where X indicates all amino acids. Mutants of non-cleavable PC1 were generated by site-specific mutagenesis to confirm cleavage at the GPS domain and to investigate the role of consensus HLT sequences for cleavage (Fig. 4A). Such a mechanism of *cis*-autoproteolysis requires Thr, Ser, and Cys, which contain nucleophilic side chains (–OH or –SH group) to support cleavage. Substitution of Thr by Ser or Cys did not disrupt the cleavage. In contrast, substitution of Thr by Val, Gly, or Arg and deletion of the GPS domain (ΔGPS) blocked the cleavage. To confirm the correlation between cleavage and pathologic mutations affecting the sequence at or near GPS, two germline mutants (L2993P and Q3016R) were examined. Each of the mutants almost completely inhibited the cleavage (Fig. 4B, Supplementary Fig. 12). Next, we investigated whether the activation of TRPC4β depended on PC1 cleavage at the GPS site. Non-cleavable mutants (L2993P, Q3016R and T3049G/R/V) did not activate TRPC4β channels. In contrast, TRPC4β currents were increased in cells transfected with T3049C or T3049S, where cleavage occurs at the GPS (Fig. 4C). These results suggest that cleavage at the HL↓T of PC1 is required for the activation of TRPC4 channels.

### STAT1 phosphorylation by PC1-mediated activity of TRPC4β

**Figure 5:**
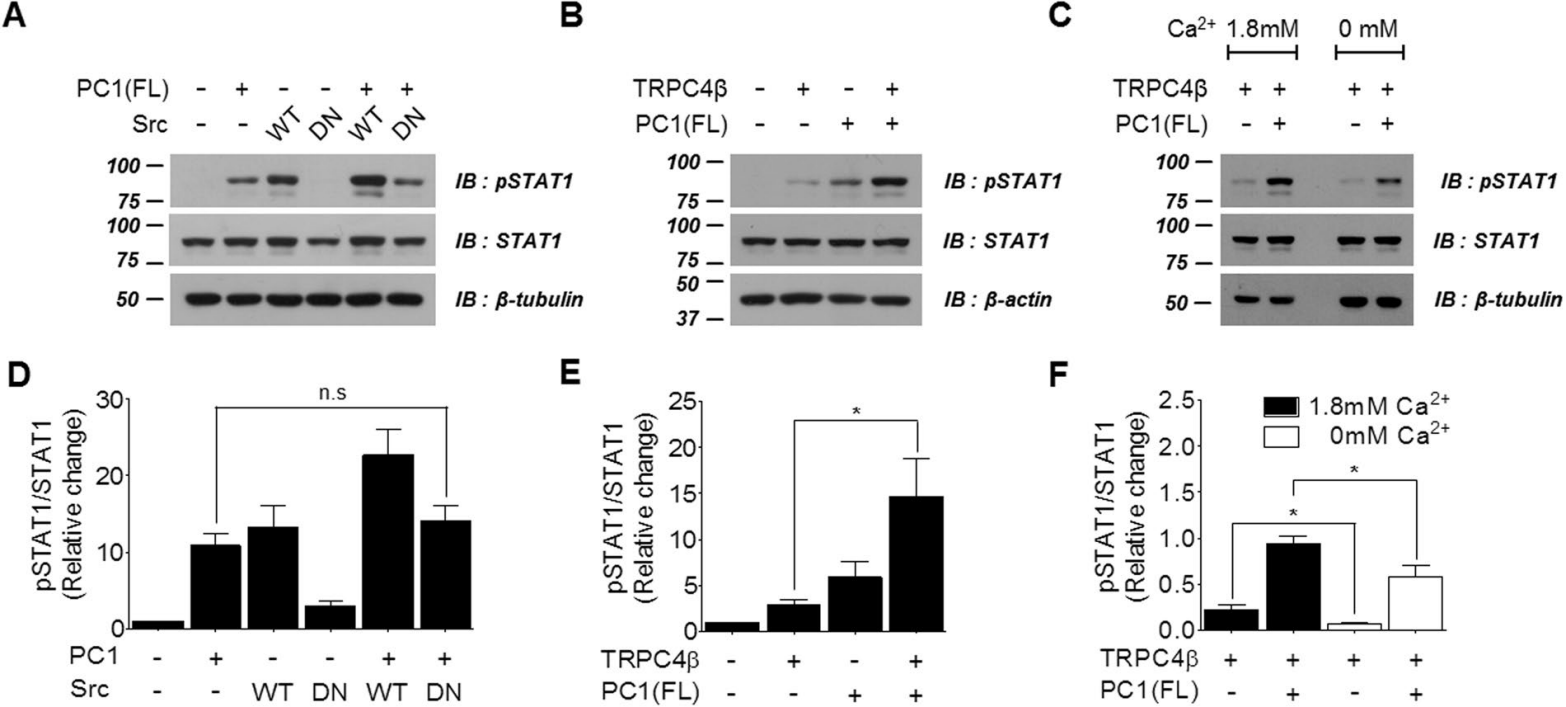
STAT1 phosphorylation by PC1-mediated activity of TRPC4β. (**A**) Effects of Src kinase on STAT1 phosphorylation. HEK 293 cells were transfected with PC1(FL) and wild-type or dominant-negative Src. Cell lysates were used for Western blot analysis with antibodies against total and phosphorylated STAT1. (**B**) STAT1 phosphorylation by PC1-mediated TRPC4β signaling. HEK 293 cells were transfected with PC1(FL) and/or TRPC4β. Levels of pSTAT1 and STAT1 were assessed by Western blotting. (**C**) Effects of Ca^2+^ on STAT1 phosphorylation by PC1/TRPC4β. HEK 293 cells were transfected or co-transfected with TRPC4β and PC1(FL). After transfection, extracellular calcium ions were removed using Ca^2+^-free medium. Levels of pSTAT1 and STAT1 were assessed by Western blotting. (**D**–**F)** Bar graphs showing mean levels of phosphorylated STAT1 relative to total STAT1 protein levels. Statistical significance is denoted by an asterisk (*p < 0.05).

PC1 has been implicated in a variety of intracellular signaling events, including JAK-STAT signaling. Overexpression of full-length PC1 can activate signal transducer and activator of transcription 1 (STAT1), STAT3 and STAT6^9,31,32^, which mediate signaling involved in proliferation, differentiation and death. STATs are phosphorylated and activated by protein tyrosine kinases, including growth factor receptors such as EGFR, and non-receptor tyrosine kinases (e.g., Src and JAK)^33^. To confirm whether STAT1 activation by PC1 was dependently regulated by Src kinase activity, we transiently transfected HEK 293 cells with dominant-negative Src (Src^DN^) mutants. The basal level of phosphorylated STAT1 was not detected in HEK cells or changed by Src^DN^ expression. STAT1 was activated by PC1 and wild-type Src (Src^WT^), and co-expression of PC1 and Src further increased STAT1 activation levels. Activation of STAT1 by PC1 was not affected by Src^DN^ (Fig. 5A,D, Supplementary Fig. 13). These results suggest that PC1 leads to Src-independent activation of STAT1 through tyrosine phosphorylation.

We have also investigated whether activation of the TRPC4β channel by PC1 affects STAT1 phosphorylation. STAT1 was not activated by the TRPC4β channel itself. Activation of STAT1 was increased by PC1-mediated activation of TRPC4β when TRPC4β was co-expressed with PC-1 (Fig. 5B,E, Supplementary Fig. 13). Since it has been reported that CTT of PC1 is released by γ-secretase-mediated cleavage^34^, we investigated whether the phosphorylation levels of STAT1 through PC1-mediated TRPC4β activity was inhibited by the γ-secretase inhibitor DAPT (Supplementary Fig. 4). Transfection mixtures of PC1 and/or TRPC4β were added drop-wise to cell culture media containing 80 μmol/L DAPT and incubated for 24 hours. STAT1 activity of PC1/TRPC4β was inhibited by DAPT, indicating that STAT1 phosphorylation is associated with CTT cleaved from PC1. To investigate the potential role of Ca^2+^ entry through the TRPC4β channel in increased STAT1 phosphorylation, we observed the effects of removing extracellular calcium ions using Ca^2+^-free media. The increased level of STAT1 was significantly attenuated in Ca^2+^-free medium compared with normal conditions (Fig. 5C,F, Supplementary Fig. 13). These results suggest that the CTT of PC1 activates TRPC4β via Gα_i3_ protein and that calcium influx through TRPC4β is required for the activation of STAT1.

### Effects of the PC1/TRPC4β/STAT1 pathway on endothelial migration

**Figure 6:**
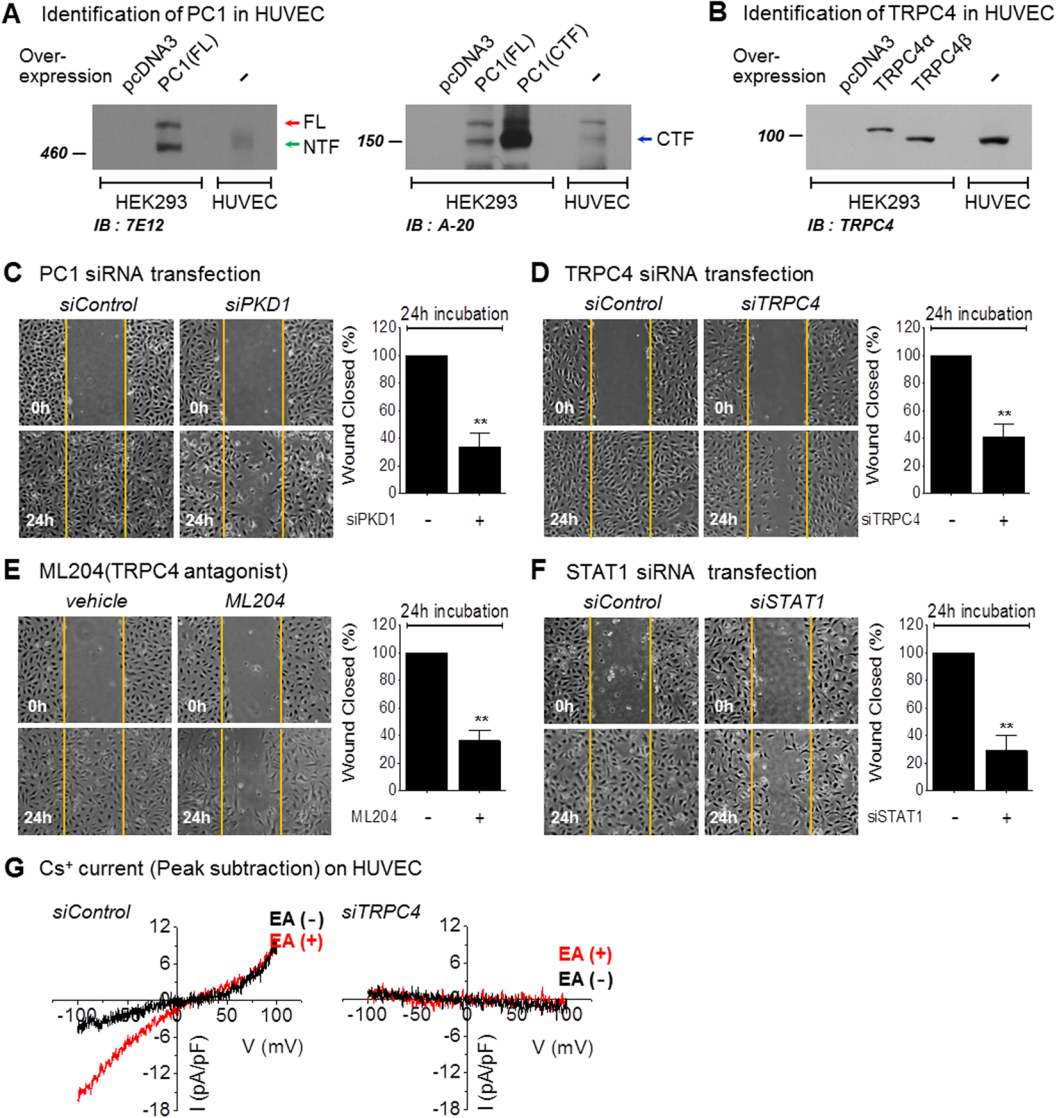
Effects of PC1 and TRPC4β on HUVECs. (**A**) Expression of PC1 in HUVECs. HEK293 cells with or without transient transfection of hPC1(FL)-flag were immunoblotted with an anti-PC1. Endogenous PC1 in HEK 293 cells and HUVECs was detected by an anti-PC1 antibody (7E12) (left panel). HEK 293 cells with or without transient transfection of hPC1(FL)-flag and HA-hPC1(CTF)-flag were immunoblotted with an anti-PC1 antibody (A-20) (right panel). Cleavage of endogenous PC1 in HUVECs; NFT (green arrow) and CTF (blue arrow) were detected. (**B**) Expression of TRPC4 in HUVECs. HEK293 cells with or without transient transfection of TRPC4α or TRPC4β were immunoblotted with an anti-TRPC4 antibody. Endogenous TRPC4β in HUVECs was detected by an anti-TRPC4 antibody. **(C)** Effects of PKD1 gene silencing on the migration of HUVECs. Wound healing assays were performed on HUVECs with siPKD1 transfection. (**D**) Effects of TRPC4 gene silencing on the migration of HUVECs. Wound healing assays were performed on HUVECs with siTRPC4 transfection. (**E**) Effects of ML204 treatment on the migration of HUVECs. Wound healing assays were performed on HUVECs in the absence or presence of the TRPC4 blocker ML204 (20 μmol/L). (**F**) Effects of STAT1 gene silencing on the migration of HUVECs. Wound healing assays were performed on HUVECs with siSTAT1 transfection. Bar graphs represent quantitative data for the cell migration assays. The area of the wound was measured at the two indicated time points in every group, and the % reduction of the initial scratch area was compared. Yellow indicates the boundary lines of the scratch. Cell migration was assessed by recovery of the scratch. (**G**) TRPC4 current activity with knock-down of TRPC4 in HUVECs. The I-V relationship is shown before (black) and after (red) Englerin A (EA) treatment. **p < 0.01, *p < 0.05 and n.s. not significant.

The TRPC family plays a role in normal and pathophysiological vascular functions^35,36^. TRPCs are involved in vascular tone (e.g., TRPC4, TRPV1, and TRPV4), regulation of vascular permeability (e.g., TRPC1, TRPC4, TRPC6, and TRPV1), hypoxia-induced vascular remodeling (e.g., TRPC4), angiogenesis (e.g., TRPC4 and TRPC6), endothelial cell proliferation, and apoptosis^37^. TRPC4 plays significant roles in normal and pathophysiological vascular function. PC1 also has an important role in vascular function. *PC1* knock-out mice died in mid-gestation with a variety of phenotypes, including a vasculopathy characterized by profound edema^38,39^. To investigate the relationship between the PC1/TRPC4β pathway and endothelial function, we first investigated the expression of TRPC4 and PC1 in HUVECs using Western blot analysis. Expression of PC1 was detected with anti-7E12 or anti-A-20 antibodies in endothelial cells (Fig. 6A, Supplementary Fig. 14). TRPC4β was predominantly expressed in endothelial cells compared with TRPC4α isoforms (Fig. 6B, Supplementary Fig. 14). In addition, Gα_i3_ was expressed in the given cell lines (Supplementary Fig. 5). These results show that all signaling proteins related to the PC1-Gα_i3_-TRPC4β pathway are expressed in HUVECs.

First, we examined the silencing effect of TRPC4β and PC1 on HUVEC migration. In wound-healing assays, endothelial cells treated with vehicle were able to close ~100% of the wound. In contrast, endothelial cells treated with 20 μmol/L ML204, a TRPC4 inhibitor, were only able to close ~40% of the wound (Fig. 6E). The migration rate of HUVECs transfected with PKD1 siRNA or TRPC4 siRNA was also significantly decreased (Fig. 6C,D). To identify whether STAT1 affects endothelial migration, STAT1 siRNA was transfected into HUVECs. The migration of STAT1-knocked-down cells was inhibited, as predicted (Fig. 6F). To test siRNA efficiency, PC1, TRPC4 and STAT1 transcript levels were evaluated by Western blot. The tested siRNAs effectively reduced the levels of protein expression (Supplementary Fig. 6). These results suggest that PC1/TRPC4β/STAT1 pathway plays an important role in endothelial migration.

To evaluate the functional activity of TRPC4, we measured the channel currents and the intracellular Ca^2+^ levels by knock-down of TRPC4. In HUVECs transfected with siControl, basal TRPC4 activity showed constitutively active current, which was induced by CsCl. Englerin A (EA), a TRPC4 agonist, slightly increased the inward current of TRPC4. Both basal and EA-evoked currents showed typical double-rectifying current-voltage relationships known to be features of the TRPC4 channel (Fig. 6G). By knock-down of TRPC4, the TRPC4-like current was completely reduced. EA-induced Ca^2+^ influx was also reduced by knock-down of TRPC4 (Fig. 6G, Supplementary Fig. 7A).

### Effects of the PC1/TRPC4β/STAT1 pathway on adherens junctions

**Figure 7:**
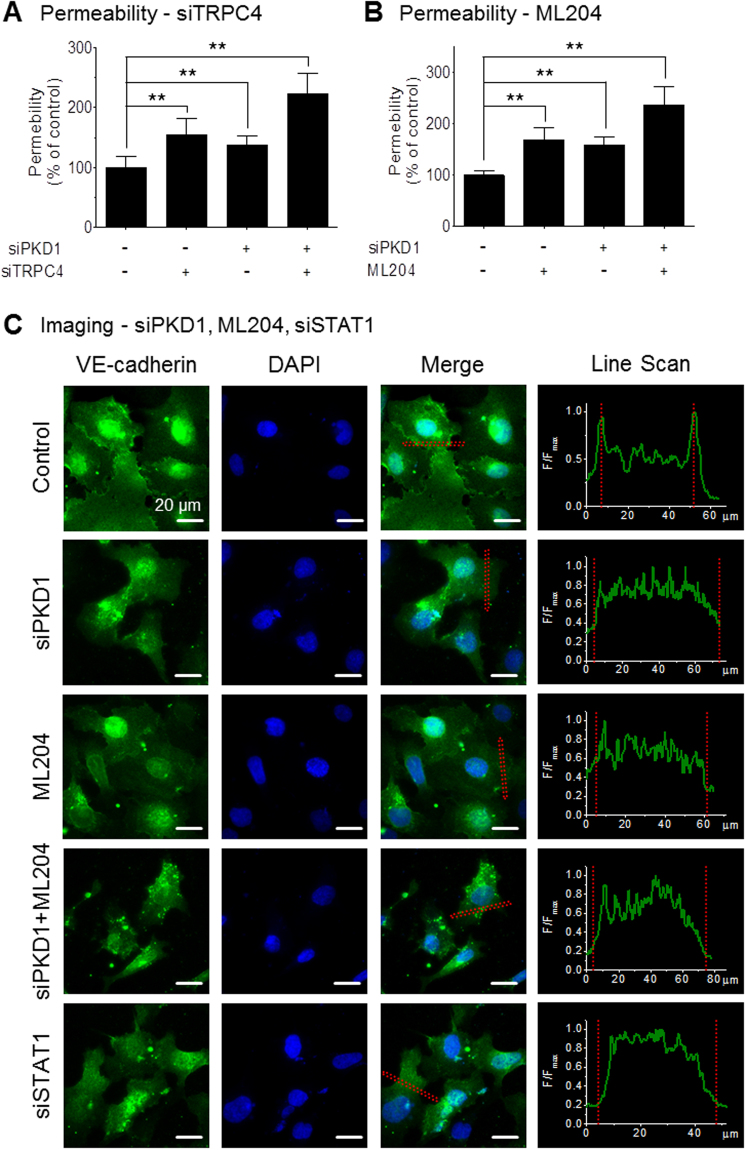
Effects of PC1 and TRPC4β on cell-cell junctions. (**A**) HUVECs were cultured in confluent monolayers on transwell membranes, and treated with siPKD1 and/or siTRPC4. (**B**) HUVECs were cultured in confluent monolayers on transwell membranes, and treated with siPKD1 and/or ML204. The permeability of the monolayers to Evans blue dye was measured by collecting media from the lower wells and measuring the absorbance at 570 nm. The down-regulation of PC1 and TRPC4 activity increased the permeability. (**C**) Localization of VE-cadherin in HUVECs treated with siPKD1, ML204 or siSTAT1. Images show a HUVEC monolayer immunolabeled for VE-cadherin (green, upper panel) and nuclei (blue, center panel), as well as the merged images (lower panel). Inhibition of PC1-mediated TRPC4β signaling disrupted the formation of cell–cell junctions. Line intensity scanning was analyzed using MetaMorph software. Statistical significance is denoted by an asterisk (**p < 0.01).

To determine whether PC1 or TRPC4β affects vascular permeability, we investigated the distribution of an endothelial-specific cadherin, VE-cadherin, and leakage of Evans blue dye in HUVECs. We evaluated the endothelial permeability using Evans blue dye. Upon siTRPC4 or ML204 treatment with or without siPKD1, the permeability of the endothelial cell monolayers to Evans blue dye was significantly increased (Fig. 7A,B). Next, the junction protein VE-cadherin was visualized via immunofluorescence staining. Treatment with siPKD1, ML204 or siSTAT1 decreased junction-localized VE-cadherin levels (Fig. 7C). Subsequently, the expression of VE-cadherin was assessed by Western blot analysis. There was no significant difference in the protein level of VE-cadherin (Supplementary Fig. 8). These data show that PC1 knock-down, ML204 or siSTAT1 treatment on endothelial cells induced VE-cadherin internalization. These results suggest that PC1-mediated TRPC4β activation plays an important role in the stability of endothelial junctions and permeability.

## Discussion

In the present study, we found that PC1-mediated TRPC4β activity plays a key role in endothelial migration and permeability. We have shown that TRPC4β can be activated by PC1, although its mechanism is not clear. Several lines of evidence suggest that PC1 functions as a G protein-coupled receptor. First, the C-terminal tail of PC1 contains a G protein-binding region. This region is conserved across many species. In our hands, PC1 coimmunoprecipitated with Gα_i3_, indicating that PC1 specifically interacts with the Gα_i3_ protein. FRET efficiency between PC1 and Gα_i3_ also increased compared with other Gα_i_ isoforms. Second, PC1 is cleaved at the GPS, which is in an immediate location of the first transmembrane domain, and this cleavage results in G protein-mediated signaling cascades. The GPS motif was first identified as a neuronal GPCR, CIRL/latrophilin^40^, and has recently been recognized as a part of the larger GPCR autoproteolysis-inducing (GAIN) domain, which is also found in PC1. Interestingly, the cleaved PC1 N-terminal fragment remains non-covalently attached to the membrane-bound C-terminal fragment^29^. Such a heterodimeric PC1 is required to transduce the signal through G proteins and plays an important role in biological functions. In contrast, it has been reported that deletion of the NTF results in constitutive activation of several aGPCRs^41^, suggesting that NTF association might normally prevent constitutive activation. Interestingly, all of the disease-associated missense mutations located in the GAIN domain and the adjacent REJ module of PC1 analyzed to date impair or disrupt cleavage. Defective GPS cleavage of PC1 has been found in a subset of ADPKD patients with aneurysmal rupture^42^. We generated non-cleavable mutants of PC1 and identified the loss of functional properties of PC1 in activating the TRPC4β channel through the Gα protein. Third, PC1 is a large integral membrane protein with 11 transmembrane segments that structurally resembles a receptor or adhesion molecule. Thus, these multiple lines of evidence demonstrate that PC1 represents structural or functional characteristics of GPCR.

In a previous report, we showed that Gα_i_ proteins played an essential and novel role in the activation of TRPC4^22^. In our hands, PC1 activated the TRPC4β channel through the Gα protein. First, PC1(FL) but not PC1(CTF) activated TRPC4β. PC1(CTF) alone did not activate TRPC4β because NTF is required for G protein-mediated signaling through the C-terminal tail of PC1. Second, non-cleavable mutants of PC1 did not activate TRPC4β current when cleavage of PC1 N-terminus and C-terminus was blocked by missense mutations at the GPS domain. Third, when intracellular Ca^2+^ levels were measured using Fura-2, PC1(FL) increased Ca^2+^ influx through the TRPC4β channel. Fourth, intracellular 0.2 mmol/L GTPγS-induced TRPC4β activation was not significantly different in the presence or absence of PC1. Fifth, activation of TRPC4β by PC1(FL) was inhibited by expression of a dominant-negative Gα_i3_ variant. Therefore, these results demonstrate unambiguously that TRPC4β is activated by an atypical Gα_i3_ coupled-receptor, PC1.

Next, we identified intracellular signaling cascades through a rise in cytosolic Ca^2+^ due to PC1/Gα_i3_/TRPC4β. Many studies have reported that disturbances in the balance between cell proliferation and apoptosis cause ADPKD. Abnormal proliferation in tubular epithelial cells plays a crucial role in cyst development and/or growth in PKD^43^. The kidneys from patients with ADPKD demonstrate high levels of apoptosis, as well as cellular proliferation^44^. Intracranial aneurysm is also believed to develop as a result of disruption of the balance between cell proliferation and apoptosis^45^. Indeed, proliferation and apoptosis must be tightly regulated. Among many regulatory factors of proliferation and apoptosis, we observed activation of STAT1 by PC1/Gα_i3_/TRPC4β. STAT1 is predominantly phosphorylated by activation of PC1-mediated TRPC4β. The Ca^2+^ influx can also lead to phosphorylation of STAT1 on tyrosine residues.

PC1, which is localized to the primary cilium, functions as flow-sensitive mechanosensor^46^. Cleavage of the PC1 protein is a response to various stimuli, such as extracellular fluid flow^47^. The products of these cleavages perform important physiological functions. Nevertheless, it is still unclear how and where PC1 is cleaved. According to previously reported data, there are cleavage-inducible factors, such as mechanical stimuli^47^, polycystin-2 (PC2)^48^, and γ-secretase^34^. Thus, perfusion flow in calcium imaging and patch clamp experiments may generate mechanosensory stimulation. In our hands, the GPS domain was used to induce autoproteolysis of PC1 into NTF and CTF, whereas the CTT of PC1 was released by γ-secretase-mediated cleavage. The increased levels of STAT1 phosphorylation via PC1-mediated TRPC4β activity were inhibited by treatment with the γ-secretase inhibitor DAPT (Supplementary Fig. 4). Thus, CTT of PC1 activated TRPC4β via the Gα_i3_ protein, and extracellular calcium through TRPC4β was required for the activation of STAT1.

Endothelial dysfunction is a hallmark of aneurysm. Aneurysm is one of the most common manifestations in ADPKD. The dysregulation of TRPC4 results in vascular endothelial dysfunction. A role of TRPC4 in vascular endothelial function was previously reported that acetylcholine-induced vasorelaxation occurred due to acetylcholine-dependent NO production in aortic endothelial cells, which was largely reduced in TRPC4 knock-out mice^49^. Reduced calcium entry in TRPC4-deficient lung endothelial cells was also associated with reduced thrombin-induced formation of actin stress fibers, reduced endothelial cell retraction, and reduced microvascular endothelial permeability evoked by a PAR-1 agonist peptide in isolated lungs^50^. A missense SNP (TRPC4-I957V) in the TRPC4 human gene was further found to be associated with a reduced risk of myocardial infarction. The proposed mechanism underlying protection involved improved endothelial function^51^. In addition, a TRPC4-mediated Ca^2+^ signaling pathway evoked by EGF was specifically identified in sub-confluent, proliferating clusters of human microvascular endothelial cells. The abundance of TRPC4 in the plasma membrane and its contribution to Ca^2+^ entry depend on the proliferation state, and its activity is regulated by cell–cell contact formation in a β-catenin-dependent manner^23^. In our hands, inhibition of PC1-mediated TRPC4β activity induced endothelial dysfunction, as demonstrated by the reduction of endothelial migration (Fig. 6C–E) and cell-cell junctions (Fig. 7C) by down-regulation of PC1 or TRPC4. Although histamine and thrombin could increase the permeability of HUVECs by a robust Ca^2+^ release and Ca^2+^ entry (Supplementary Fig. 7) via receptor-operated and store-operated channel (SOC), sustained Ca^2+^ influx through TRPC4, which is constitutively activated by PC1, may play a role in refilling the ER Ca^2+^ stores, which require further investigation. Furthermore, Ca^2+^-dependent STAT1 participates in other signaling pathways associated with endothelial function in strengthening tight junctions. Thus, knock-down of PKD1, TRPC4 or STAT1 increases endothelial permeability. Based on these findings, we propose the activation of PC1-mediated TRPC4β in endothelial cells as a tentative molecular target for cerebral aneurysms associated with ADPKD.

**Figure 8:**
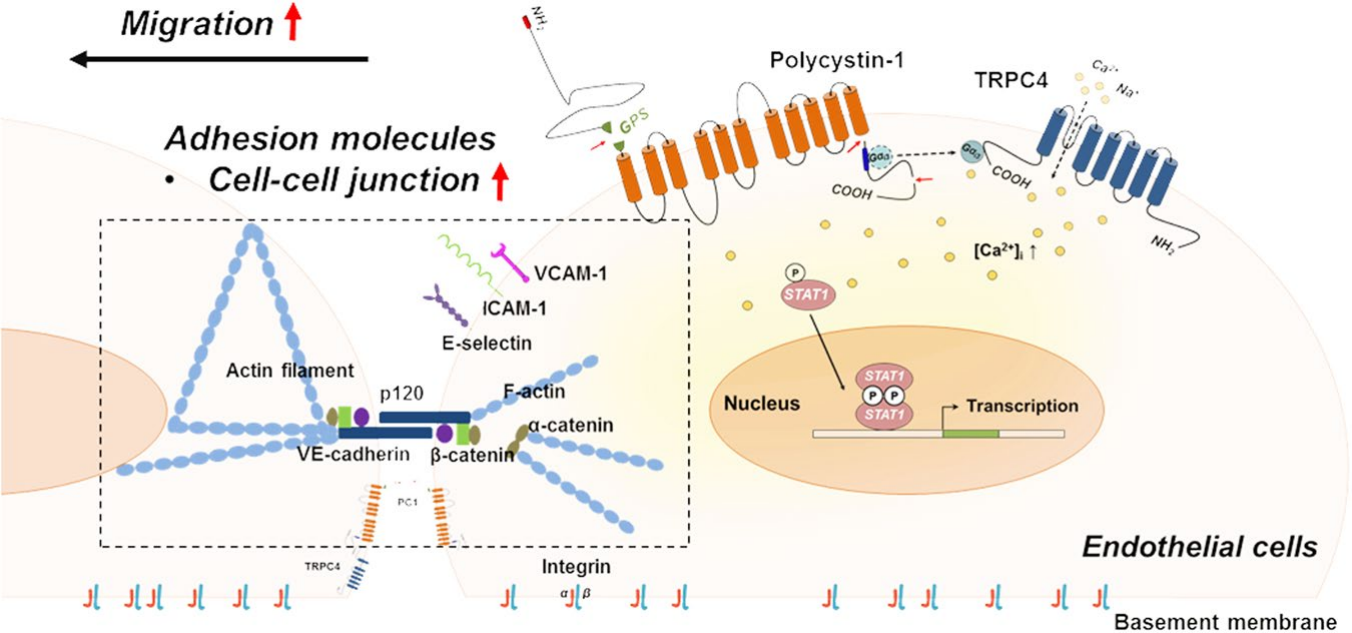
Schematic summary of the proposed model.

In conclusion, the main findings of this study are as follows (Fig. 8): (1) PC1(FL) but not PC1(CTF) activates TRPC4β via Gα_i3_. (2) PC1 and TRPC4β increase Ca^2+^-dependent STAT1 activation. (3) The down-regulation of PC1 and TRPC4β activity reduces endothelial cell migration. (4) Knock-down of STAT1 reduces endothelial cell migration. (5) Inhibition of PC1-mediated TRPC4β signaling increases endothelial cell permeability by disruption of cell-cell junctions. Thus, mutations of *PKD1* contribute to endothelial dysfunction via decreased migration and increased endothelial permeability.

## Materials and Methods

### Cell culture, transient transfection, and chemicals

Human embryonic kidney (HEK)-293 cells (American Type Culture Collection, USA) were maintained in Dulbecco’s modified Eagle’s medium (HyClone, USA) supplemented with 10% fetal bovine serum (FBS), 100 U/mL penicillin, and 100 μg/mL streptomycin according to the supplier’s recommendations. Human umbilical vein endothelial cells (HUVECs) were cultured in M199 medium (Welgene, South Korea) containing 20% FBS, 3 ng/ml human FGF-basic (Peprotech, USA), and antibiotics. Prior to transient transfection, cells were seeded in 6- or 12-well plates. The following day, 0.5–2 μg/well TRPC4β and PKD1 cDNA was transfected into cells using the transfection reagent FuGENE 6 (Roche Molecular Biochemicals, USA) for electrophysiological experiments, according to the manufacturer’s protocol. For molecular biology experiments, Lipofectamine 2000 (Invitrogen, USA) was used as the transfection reagent. All experiments were performed 20–30 hours after transfection. For gene silencing, siRNA was transfected using the transfection reagent RNAi Max (Invitrogen, USA) according to the manufacturer’s instructions. Control siRNA (Cat. SN-1003), human PKD1 siRNA (Cat. 1117253) and human TRPC4 (Cat. 1156755) were purchased from Bioneer (South Korea). Human STAT1 (Cat. sc-44123) was purchased from Santa Cruz (USA). PTX and Englerin A was purchased from Sigma Aldrich (USA).

### Plasmids

Human PKD1(FL) in pGFP-N1 and human PKD1(FL)-Flag in pCI-neo plasmids were kindly provided by Eric Honoré and Gregory Germino, respectively. HA-human PKD1(CTF)-Flag in pCI and EGFP-human PKD1(FL) in pCI were kindly provided by Feng Qian (Johns Hopkins University). Human PKD1(FL) was subcloned into the pECFP-N1 and pEYFP-N1 vectors.

### Western blotting, Co-IP, and Surface biotinylation

Cells were plated in 6-well dishes. Lysates were prepared in lysis buffer (0.5% Triton X-100, 150 mmol/L NaCl, 50 mmol/L HEPES, 2 mmol/L MgCl_2_, 2 mmol/L EDTA, pH 7.4) via passage 10–15 times through a 26-gauge needle after sonication. After lysates were centrifuged at 13,000 × g for 10 minutes at 4 °C, the protein concentration in the supernatants was determined. The extracted proteins in sample buffer were loaded onto 5, 8, or 10% Tris-glycine sodium dodecyl sulfate polyacrylamide gel electrophoresis (SDS-PAGE) gels. The proteins were transferred onto a PVDF membrane. For details concerning the antibodies, please see the supplementary materials.

In the Co-IP experiments for detection of PC1-Gα subtypes, 500 μL of cell lysates (500–1000 μg) were incubated with 1 μg of anti-PKD1 (A-20) or anti-Gα antibodies and 30 μL of protein G-agarose beads at 4 °C overnight with gentle rotation. After the beads were washed three times with wash buffer (0.1% Triton X-100), the precipitates were then eluted with 30 μL of 2× Laemmli buffer and subjected to Western blot analysis.

For surface biotinylation, cells were first washed with PBS and incubated in 0.5 mg/mL sulfo-NHS-LC-biotin (Pierce, USA) in PBS for 30 minutes on ice. The biotin was quenched by 100 mmol/L glycine in PBS. The cells were then processed as described above for cell extraction. Forty microliters of 1:1 slurry of immobilized avidin beads (Pierce, USA) was added to 300 μL of cell lysates (500 μg protein). After incubation for 1 hour at room temperature, the beads were washed three times with 0.5% Triton X-100 in PBS, and proteins were extracted in sample buffer. The collected proteins were analyzed by Western blot.

### Electrophysiology

The transfected cells were trypsinized and transferred to a recording chamber, which was equipped for the application of a number of solutions. Whole-cell currents were recorded using an Axopatch 200B amplifier (Axon Instruments, USA) and Digidata 1440 A Interface (Axon Instruments), and analyzed using a personal computer equipped with pClamp 10.2 software (Axon Instruments) and Origin software (Microcal origin v.8.0, USA). Patch pipettes were made from borosilicate glass and had resistances of 2–4 MΩ when filled with standard intracellular solutions. For whole cell experiments, we used an external bath medium (normal Tyrode solution) of the following composition (in mmol/L): 135 NaCl, 5 KCl, 2 CaCl_2_, 1 MgCl_2_, 10 glucose, and 10 *N*-[2-hydroxyethyl]piperazine-*N*′-[2-ethanesulfonic acid] (HEPES) with the pH adjusted to 7.4 using NaOH. Cs^+^-rich external solution was made by replacing NaCl and KCl with equimolar CsCl. The standard pipette solution contained the following (in mmol/L): 140 CsCl, 10 HEPES, 0.2 Tris-GTP, 0.5 EGTA, and 3 Mg-ATP with the pH adjusted to 7.3 using CsOH. Intracellular 50 nmol/L, 200 nmol/L, or 5 μmol/L free Ca^2+^ pipette solutions were chosen on the basis of previous studies of TRPC5. Voltage ramp pulses were applied at −60 mV with a holding potential from +100 to −100 mV for 500 ms. A salt-agar bridge was used to connect the ground Ag-AgCl wire to the bath solution for the experiments that used reducing agents. All current traces are the selected values at −60 or +80 mV of the ramp pulses. The inward current amplitudes at −60 mV are summarized for all bar graphs. The current recording was performed as previously described^52^.

### Fluorescence Resonance Energy Transfer (FRET) measurements

Three FRET images (cube settings for CFP, YFP, and Raw FRET) were obtained from a pE-1 Main Unit to 3 FRET cubes (excitation, dichroic mirror, filter) via a fixed collimator. The excitation LED and the filter were sequentially rotated, and the rotation period for each filter cube was ~0.5 s. All of the images were obtained within 1.5 seconds. Each image was captured on a cooled 10 MHz (14 bit) CCD camera (ANDOR technology, USA) with 100 ms of exposure time with 2 × 2 binning (645 × 519 pixels). Using IX70, an Olympus microscope equipped with a 60× oil objective, the three-cube FRET efficiency was analyzed using MetaMorph 7.6 software (Molecular Devices, USA). For details of FRET ratio and FRET efficiency computation, please see the supplementary materials.

### Intracellular Ca^2+^ measurements with Fura-2

The ratiometric measurement of [Ca^2+^]_i_ was performed using Fura-2-AM (molecular probe, USA). The cells were seeded in 24-well dishes and loaded with 5 μmol/L of Fura-2-AM for 30 minutes at 37 °C. The Fura-2 fluorescence was measured at 510 nm emission with 340/380 nm dual excitation using a DG-4 illuminator. The experiments were performed in a normal solution containing 145 mmol/L NaCl, 3.6 mmol/L KCl, 10 mmol/L HEPES, 1.3 mmol/L CaCl_2_, 1 mmol/L MgCl_2_, and 5 mmol/L glucose with pH adjusted to 7.4 using NaOH.

### Wound-healing assay

HUVECs transfected with PKD1, TRPC4 or STAT1 siRNA were seeded in 6 well culture plates and incubated with ML204, a selective TRPC4 channel inhibitor. Cells were grown as high-density monolayers, scratched with a 200 μL pipette tip, and allowed to migrate for the indicated times after three washes to remove detached cells. Migration was recorded using a Nikon ECLIPSE TS100 microscope equipped with a Lumenera’s INFINITY1-3 digital camera. The area covered by the monolayer was measured using ImageJ (National Institutes of Health, USA). All migration assays are representative of at least three independent experiments.

### *In vitro* endothelial permeability assay

To measure permeability changes of endothelial cells *in vitro*, HUVECs were cultured to confluence on cell culture inserts (3.0 μm, BD Biosciences) placed in a 12-well plate. 5 × 10^5^ HUVECs were seeded on each cell culture insert. In control samples, 500 μL of Evans blue dye (0.67 mg/mL) was added to the upper compartment to demonstrate that the endothelial layers were impermeable to this dye. Experimental wells were treated with siPKD1, siTRPC4 or ML204 for 48 hours, and 500 μL dye was added to the upper compartments. The liquid was collected from the lower wells after 10 minutes and measured by spectrophotometric absorbance at 570 nm.

### Immunofluorescence microscopy

HUVECs were treated with siPKD1, ML204 or siSTAT1 for 48 hours. The cells were fixed with 4% paraformaldehyde for 15 minutes. After washing in PBS, cells were incubated with an anti–VE-cadherin antibody at 4 °C overnight. VE-cadherin was localized using Alexa Fluor 488 (Invitrogen, USA). Nuclei were stained with Hoechst 33342 (Invitrogen, USA). The cells were examined using a fluorescence microscope, and images were analyzed using Metamorph software (Molecular Devices, USA).

### Statistics

The results are presented as the means ± s.e.m. They were compared using Student’s t-tests between two groups, or ANOVA followed by post-hoc tests among three or more groups. p < 0.05 was considered statistically significant. The number of cells for electrical recordings is provided as an n value in the bar graphs.

### Data availability

The data generated during the current study are available from the corresponding author upon reasonable request.

## Electronic supplementary material

Supplementary information
