# Supplementary material for: Gαi-mediated TRPC4 activation by polycystin-1 contributes to endothelial function via STAT1 activation
Source: Sci Rep. 2018 Feb 22;8:3480. doi: 10.1038/s41598-018-21873-1 (PMC5823873; doi:10.1038/s41598-018-21873-1)
Supplement: Supplementary file 1 — Supplementary information [file 41598_2018_21873_MOESM1_ESM.docx]

**Supplementary information**

**Gα_i_-mediated TRPC4 activation by polycystin-1 contributes to the endothelial function via STAT1 activation.**

Misun Kwak^1,2,#^, Chansik Hong^3,#^, Jongyun Myeong^1,2^, Eunice Yon June Park^1,2^, Ju-Hong Jeon^1,2^, Insuk So^1,2*^

^1^Department of Physiology and Institute of Dermatological Science, Seoul National University College of Medicine, Seoul 110-799, South Korea.

^2^Department of Biomedicines, Seoul National University College of Medicine, Seoul 110-799, South Korea.

^3^Department of Physiology, School of Medicine, Chosun University, Gwangju 61452, South Korea.

^#^contributed equally to the work presented here and should therefore be regarded as equivalent authors

*Corresponding Authors:

Insuk So, M.D, Ph.D.

Department of Physiology

Seoul National University College of Medicine,

28 Yeongeon-dong, Jongno-gu, Seoul 110-799, South Korea

Tel: +82-2-740-8228

insuk@snu.ac.kr

**Supplementary Figures and Figure legends**


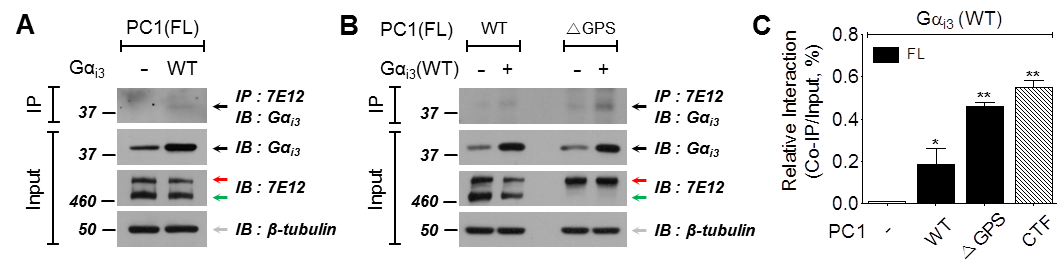


**Supplementary Figure 1.** Interaction of PC1 (FL) or PC1 (ΔGPS) with Gα_i3_ protein. **(A)** Interaction of PC1 (FL) with Gα_i3_ protein. Gα_i3_ subtype and PC1 (FL) were co-expressed in HEK 293 cells. 500 μg of proteins from each condition were subjected to immunoprecipitation with anti-7E12 and probed with an antibody against the Gα_i3_ protein. PC1 (FL) interacts directly with Gα_i3_. **(B)** Interaction of a GPS domain-deleted PC1 mutant (ΔGPS) with the Gα_i3_ protein. Gα_i3_ subtype and PC1 (ΔGPS) were co-expressed in HEK 293 cells. 500 μg of proteins from each condition were subjected to immunoprecipitation with anti-7E12 and probed with an antibody against the Gα_i3_ protein. PC1 (ΔGPS) also interacts directly with Gα_i3_. **(C)** Summary graph quantifying the relative interaction between PC1 and Gα_i3_. CTF binds with Gα_i3_ most strongly.


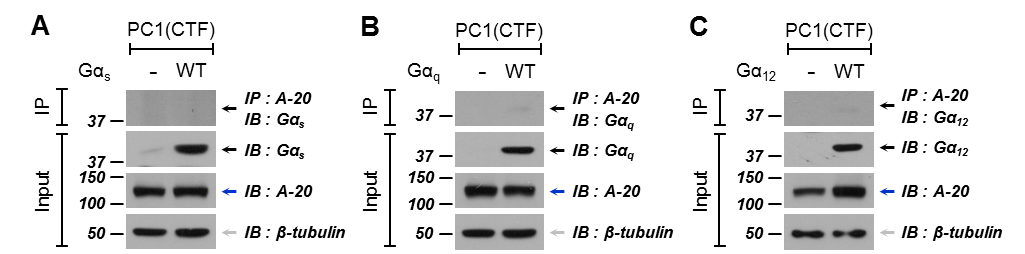


**Supplementary Figure 2.** Interaction of PC1 (CTF) with other G proteins. Gα subtypes (**(A)** Gα_s_, **(B)** Gα_q_, and **(C)** Gα_12_) and PC1 (CTF) were co-expressed in HEK 293 cells. 500 μg of proteins from each condition were subjected to immunoprecipitation with anti-A-20 and probed with an antibody against Gα proteins. PC1 (CTF) interacts directly with Gα_i3_ but not with the other Gα subtypes.


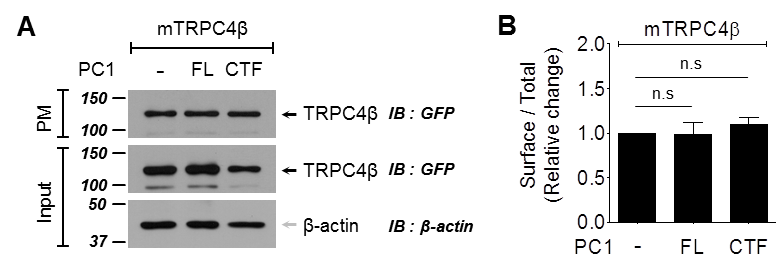


**Supplementary Figure 3.** Surface expression of TRPC4β with PC1. **(A)** Representative cell surface biotinylation assay showing surface (PM) and total expression of TRPC4β, as indicated, after co-expression with PC1(FL) or PC1(CTF). Biotinylated and total cellular proteins were quantified by immunoblot analysis. Surface expression of TRPC4β with PC1 was not altered. **(B)** Bar graph showing densitometric analyses of 3 repetitions of this experiment.


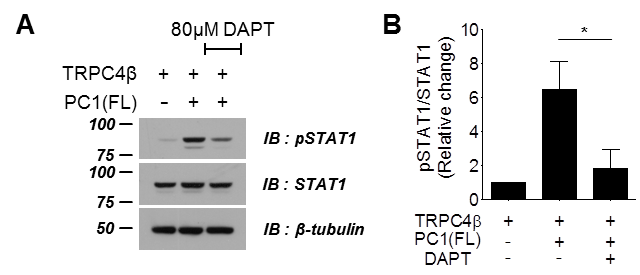


**Supplementary Figure 4.** STAT1 phosphorylation with a γ-secretase inhibitor. **(A)** HEK 293 cells were transfected with PC1 (FL) and/or TRPC4β. The γ-secretase inhibitor DAPT (80 μM) was added to the media. Levels of pSTAT1 and STAT1 were assessed by Western blotting. **(B)** Quantification of Western blotting images by ImageJ. The data are representative of three experiments. Bar graph showing mean levels of phosphorylated STAT1 relative to total STAT1 protein. Statistical significance is denoted by an asterisk (*, p＜0.05).


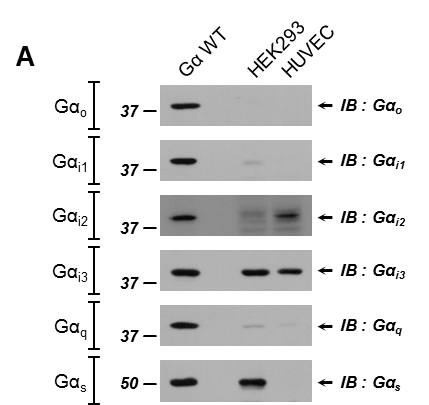


**Supplementary Figure 5.** **(A)** The expression of endogenous G proteins in HUVECs. HEK 293 cells were transfected with Gα subtypes. Levels of Gα proteins were assessed by Western blotting.


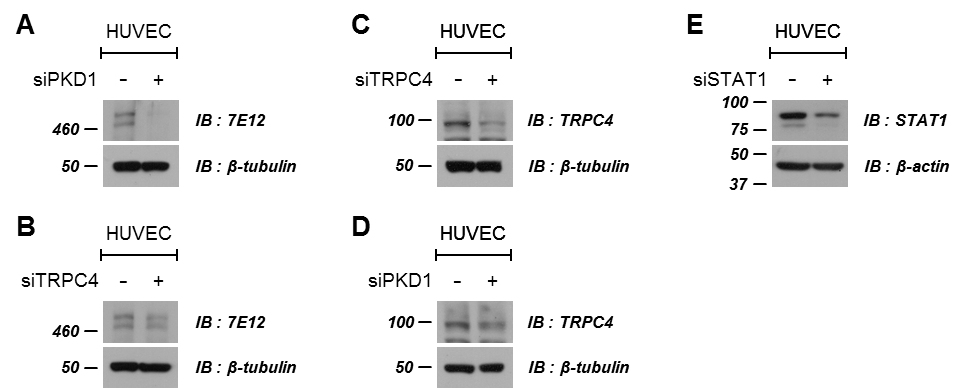


**Supplementary Figure 6.** Analysis of siRNA-mediated silencing of PKD1 **(A and B)**, TRPC4 **(C and D)**, or STAT1 **(E)** expression in HUVECs. HUVECs were transfected with siRNAs and harvested at 48 h post-transfection. Western blot analysis was performed with the indicated antibodies.


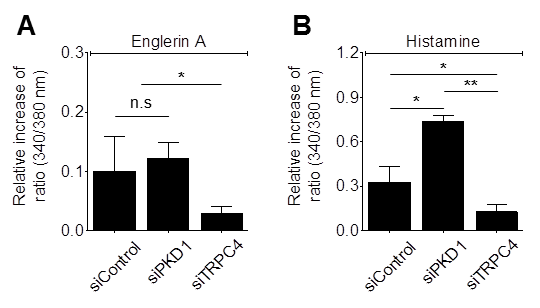


**Supplementary Figure 7.** Ca^2+^ measurement with knock-down of PKD1 or TRPC4 in HUVECs using 100 μM EA **(A)** or 10 μM histamine **(B)**. ** p < 0.01, * p＜0.05 and n.s. not significant.


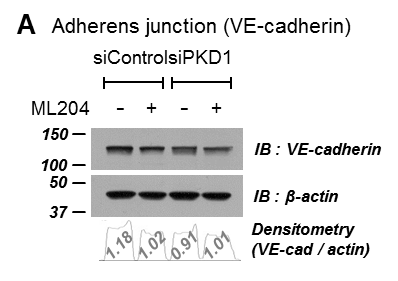


**Supplementary Figure 8. (A)** The expression of VE-cadherin in HUVECs treated with siPKD1 and/or ML204. VE-cadherin expression was detected by Western blotting. There was no significant difference in the total VE-cadherin expression. Relative VE-cadherin levels were quantified by densitometry with normalization (VE-cad /actin).


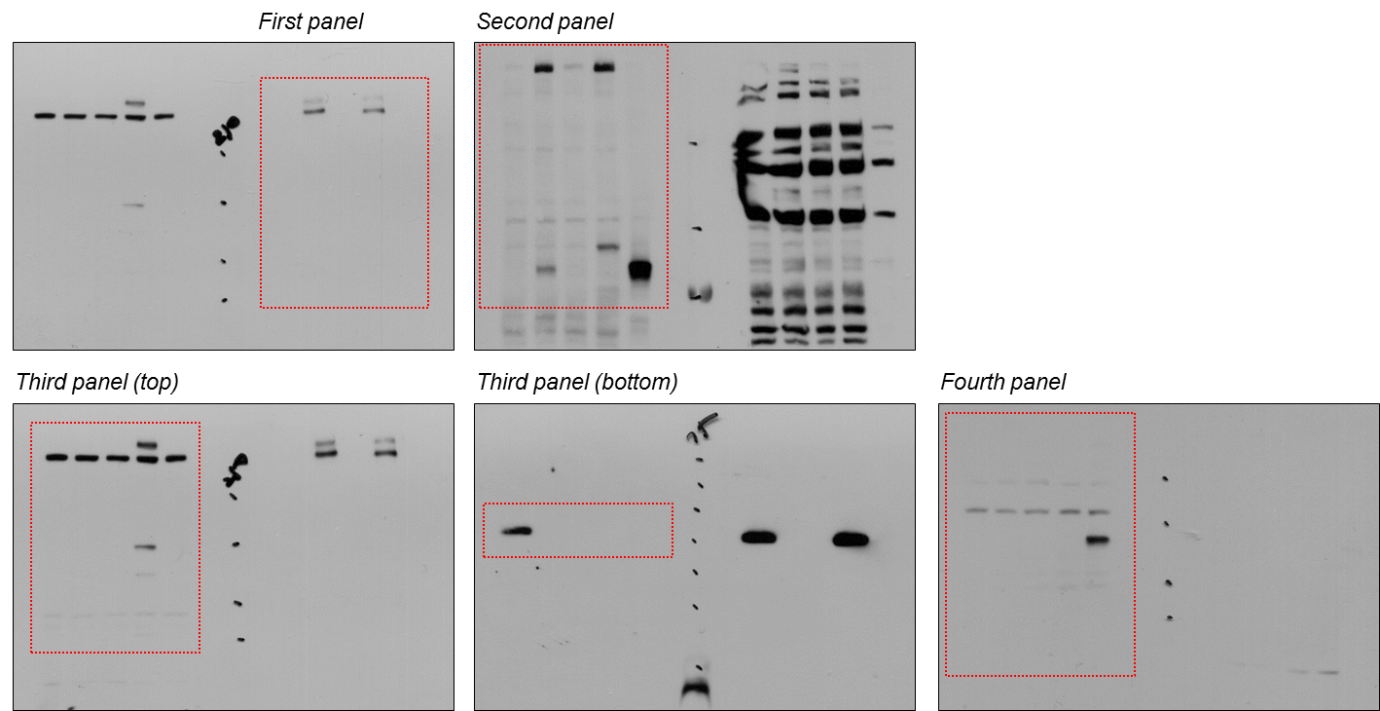


**Supplementary Figure 9.** Full blot images for Figure 1C. Red dotted lines show the cropping locations.


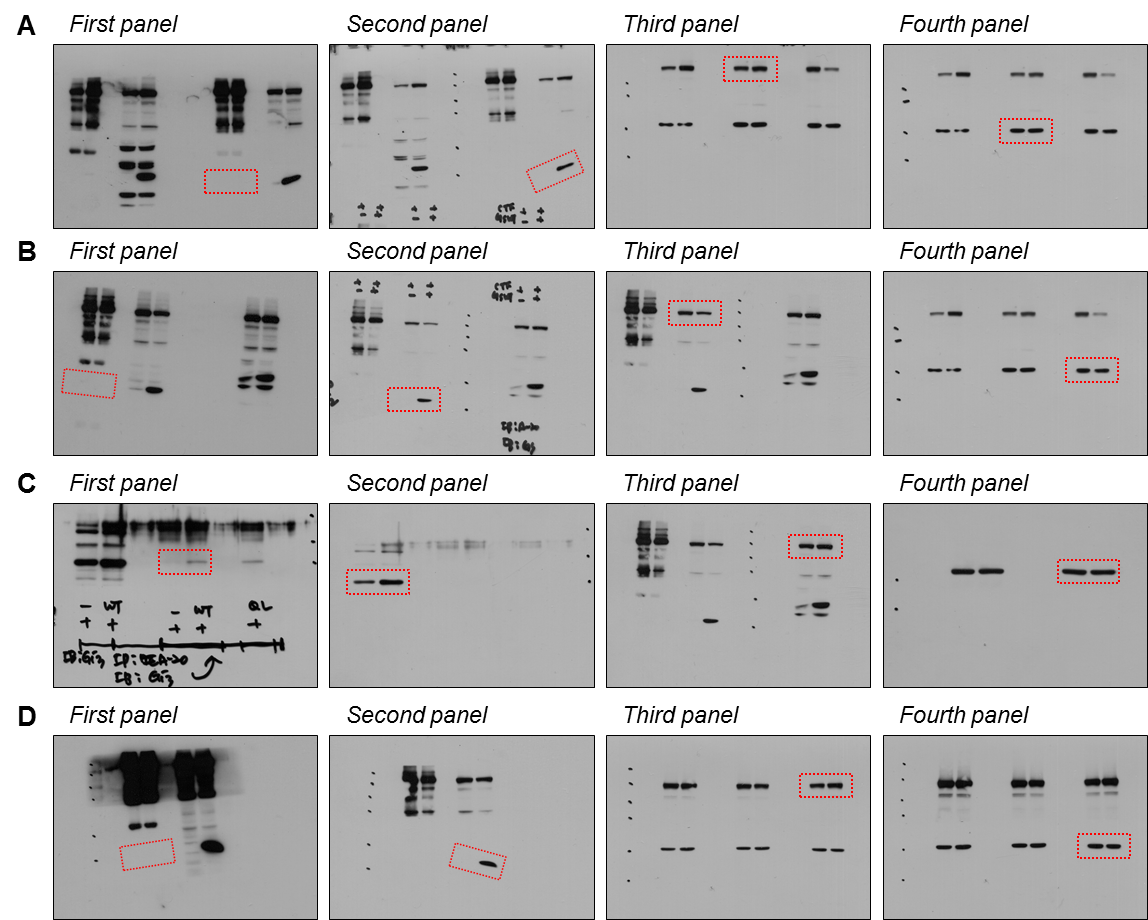


**Supplementary Figure 10.** Full blot images for Figure 2B. Red dotted lines show the cropping locations. **(A)** Full blots for the interactions between PC1(CTF) and Gα_i1._ **(B)** Full blots for the interactions between PC1(CTF) and Gα_i2._ **(C)** Full blots for the interactions between PC1(CTF) and Gα_i3._ **(D)** Full blots for the interactions between PC1(CTF) and Gα_o._


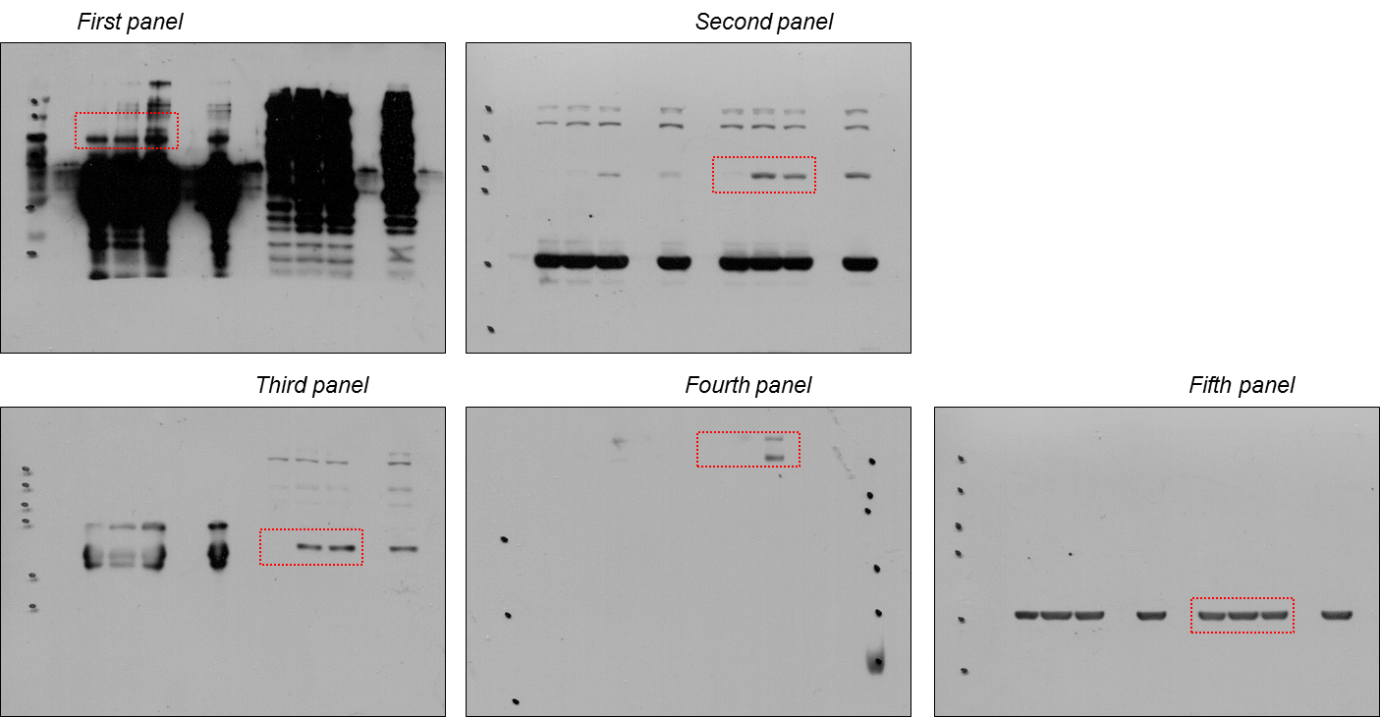


**Supplementary Figure 11.** Full blot images for Figure 3E. Red dotted lines show the cropping locations.


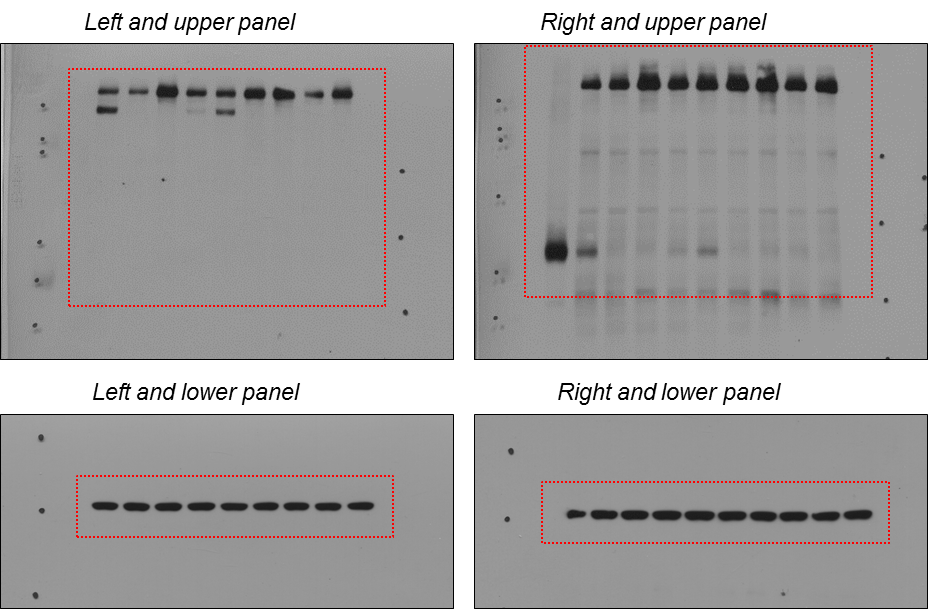


**Supplementary Figure 12.** Full blot images for Figure 4B. Red dotted lines show the cropping locations.


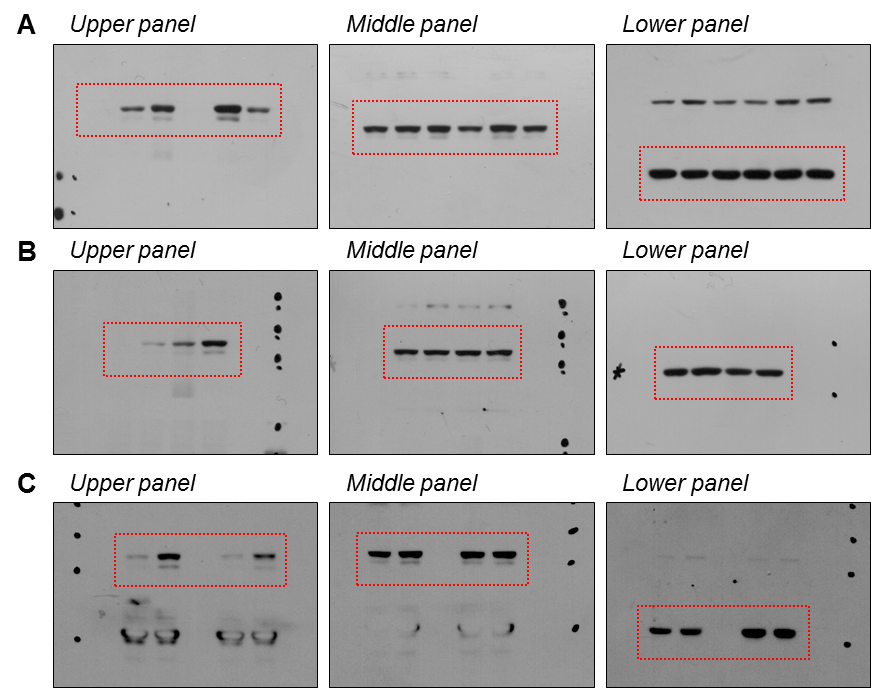


**Supplementary Figure 13.** Full blot images for Figure 5. Red dotted lines show the cropping locations. **(A)** Full blots for Fig. 5A. **(B)** Full blots for Fig. 5B. **(C)** Full blots for Fig. 5C.


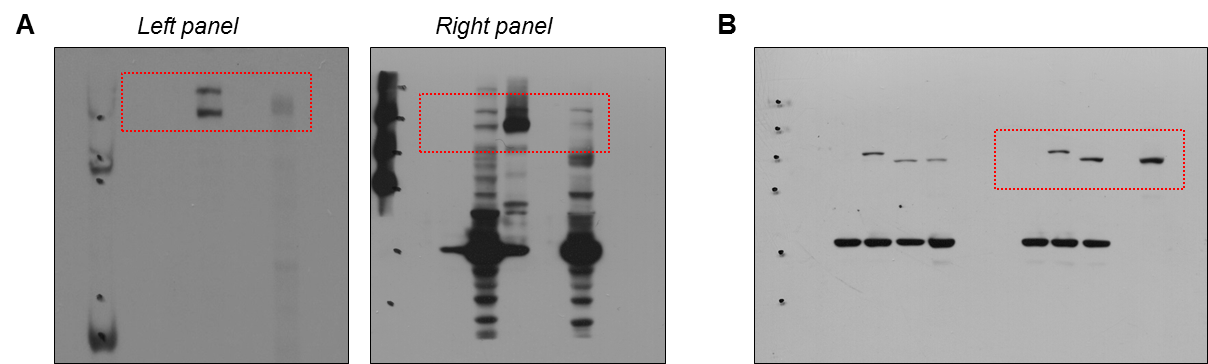


**Supplementary Figure 14.** Full blot images for Figure 6. Red dotted lines show the cropping locations. **(A)** Full blots for Fig. 6A. **(B)** Full blots for Fig. 6B.

**Supplementary Materials and Methods**

**Antibodies**

The following commercial antibodies were used: anti-PC1 (sc-130554 or sc-10371; Santa Cruz Biotechnology); anti-GFP (A11122; Life Technologies); anti-Flag (F3165; Sigma Aldrich); anti-Gα_o_ (sc-13532; Santa Cruz Biotechnology); anti-Gα_i1_ (sc-56536; Santa Cruz Biotechnology); anti-Gα_i2_ (sc-13534; Santa Cruz Biotechnology); anti-Gα_i3_ (sc-262; Santa Cruz Biotechnology); anti-Gα_o_ (sc-136181; Santa Cruz Biotechnology); anti-Gα_s_ (sc-823; Santa Cruz Biotechnology); anti-Gα_12_ (sc-409; Santa Cruz Biotechnology); anti-STAT1 (#9172; Cell Signaling); anti-p-STAT1 (#9167; Cell Signaling); anti-TRPC4 (73-119; Neuromab); anti-VE-cadherin (sc-6458; Santa Cruz Biotechnology); anti-β-actin (GTX109639; GeneTex); and anti-β-tubulin (T-4026; Sigma Aldrich).

**FRET ratio and FRET efficiency computation**

FRET Ratio (FR) can be regarded as the fractional increase in FRET-induced YFP emission and calculated as:

$$FR = \frac{F_{A_{D}}}{F_{A}} = \frac{[S_{FRET}\left( DA \right)-R_{D1}\cdot S_{CFP}\left( DA \right)]}{R_{A1}\cdot[S_{YFP}\left( DA \right)-R_{D2}\cdot S_{CFP}\left( DA \right)]}$$

where S_CUBE_ (SPECIMENDA) indicates the intensity measurement, CUBE indicates the filter cube (FRET, CFP, or YFP) and SPECIMEN indicates donor (D, CFP), acceptor (A, YFP), or both (DA). $R_{D1} ={S_{\mathrm{FRET}} \left( D \right)}/{S_{\mathrm{CFP}} (D)}$,$R_{D2}={S_{\mathrm{YFP}} (D)}/{S_{\mathrm{CFP}} (D)}$, and $R_{A1}=S_{\mathrm{FRET}} \left( A \right) /S_{\mathrm{YFP}} \left( A \right)$ are constants derived from measurements applied to single cells with only CFP- or YFP-tagged molecules. Three-cube FRET does not require that the fusion constructs of CFP and YFP preserve the spectral features of fluorophores. Similar ratios and recorded spectra provide indications that the spectral features of the fluorophores are not perturbed by fusion. Since FR relies on YFP emission, YFP is attached to the presumed limiting moiety in a given interaction. Subsequent quantitative calculations based on FR rely on a presumed 1:1 interaction stoichiometry. The effective FRET efficiency (E_EFF_) was determined as:

$$E_{\mathrm{EFF}}=E\times A_{b}=\left( FR-1 \right)[E_{\mathrm{YFP}} (440)/E_{\mathrm{CFP}} (440)]$$

where E represents the intrinsic FRET efficiency when molecules with fluorophores are associated with each other, A_b_ represents the fraction of YFP-tagged molecules that interact with CFP-tagged molecules, and the bracketed term represents the ratio of YFP and CFP molar extinction coefficients for the FRET cube excitation filter^53^. The ratio we have determined in this experiment is 0.094 based on the maximal extinction coefficients for ECFP and EYFP^54^ and the excitation spectra measured in our lab. FRET was also assessed by measuring the level of dequenching of donor emission followed by nearly complete photodestruction of the acceptor^55^ with strong illumination for 30 min through a 540AF30 excitation filter. This spared the CFP chromophore for control experiments. Here, the effective FRET efficiency was calculated as:

$$E_{\mathrm{EFF}}=E\times D_{b}=[1-{S_{\mathrm{CFP}}\left( \mathrm{DA} \right)_{\mathrm{before}}}/{S_{\mathrm{CFP}}\left( \mathrm{DA} \right)_{\mathrm{after}}]}$$

where $S_{\mathrm{CFP}} \left( \mathrm{DA} \right)_{\mathrm{before}}$ and $S_{\mathrm{CFP}}\left( \mathrm{DA} \right)_{\mathrm{after}}$ are CFP emission before and after YFP photobleaching, respectively, and D_b_ indicates the fraction of CFP-tagged molecules that interacted with YFP-tagged molecules^53^.

**Supplementary References**

1. Epe, B., Steinhäuser, K.G., Woolley, P. Theory of measurement of Förster-type energy transfer in macromolecules. *Proc Natl Acad Sci U S A* **80**(9), 2579-83 (1983).
2. Patterson, G., Day, R. N., Piston, D. Fluorescent protein spectra. *J Cell Sci* **114**(Pt 5), 837-8 (2001).
3. Miyawaki, A., Tsien, R.Y. Monitoring protein conformations and interactions by fluorescence resonance energy transfer between mutants of green fluorescent protein. *Methods Enzymol* **327**, 472-500 (2000).
